# Development and Validation of a NEWS2-Enhanced Multivariable Prediction Model for Clinical Deterioration and In-Hospital Mortality in Hospitalized Adults

**DOI:** 10.3390/medicina61091543

**Published:** 2025-08-27

**Authors:** Sofia Lo Conte, Guido Fruscoloni, Alessandra Cartocci, Martin Vitiello, Maria Francesca De Marco, Gabriele Cevenini, Paolo Barbini

**Affiliations:** 1 Unit of Diagnostic and Therapeutic Neuroradiology, Department of Neurology and Human Movement Sciences, Azienda Ospedaliero Universitaria, 53100 Siena, Italy; sofia.loconte@student.unisi.it (S.L.C.); vitiellomartin@gmail.com (M.V.); 2 Health Management Department, Azienda Ospedaliero Universitaria, 53100 Siena, Italy; g.fruscoloni@ao-siena.toscana.it (G.F.); f.demarco@ao-siena.toscana.it (M.F.D.M.); 3 Department of Medical Sciences, Surgery and Neurosciences, University of Siena, 53100 Siena, Italy; paolo.barbini@unisi.it; 4 Department of Medical Biotechnologies, University of Siena, 53100 Siena, Italy; gabriele.cevenini@unisi.it

**Keywords:** NEWS2, patients deterioration, risk stratification, clinical risk prediction, electronic health record

## Abstract

*Background and Objectives*: Early identification of patients at risk of clinical deterioration is essential for optimizing therapeutic management and improving outcomes in general medicine wards. The National Early Warning Score 2 (NEWS2) is a validated tool for predicting patient worsening but integrating it with additional clinical and demographic data can enhance its predictive accuracy and support timely clinical decisions. *Material and methods*: In this retrospective cohort study, 2108 patients admitted to the general medicine department of the University Hospital of Siena were analyzed. Logistic regression models incorporating NEWS2 alongside key clinical variables—including age, presence of central venous catheter (CVC), and functional status measured by the Barthel Index—were developed to predict high clinical risk (HCR) and mortality. Model performance was assessed using the area under the ROC curve (AUC). *Results*: High clinical risk status developed in 29% of patients. Older age, presence of CVC, lower Barthel Index, and higher NEWS2 scores were significantly associated with both HCR and mortality. The integrated predictive model demonstrated good accuracy, with an AUC of 0.798 for HCR and 0.716 for mortality prediction. *Conclusions*: This study suggests that NEWS2, when combined with additional patient-specific variables from the electronic health record, can become a more sophisticated tool for early risk stratification. Such a tool has the potential to support timely clinical intervention and optimized therapeutic management, potentially contributing to improved patient outcomes. While the model may indirectly support nurse workload balancing by identifying patients requiring intensified care, its ultimate impact on patient outcomes requires confirmation through prospective studies.

## 1. Introduction

Early recognition of clinical deterioration in hospitalized patients is essential to ensure safe and effective medical care, enabling the proper allocation of resources and improving patient outcomes [1,2]. However, early interception of a patient’s declining condition is still a challenge, especially in general medical wards, where patients have highly heterogeneous conditions [3].

Clinical deterioration often begins with imperceptible and nonspecific signs, such as slight changes in vital parameters, mild confusional state, or decreased responsiveness of the patient, which can be easily missed or misunderstood [4,5]. This is especially true in high-demand environments where the increasing workload of physicians and nurses makes the timely detection of a patient’s decline progressively more difficult [6]. This complexity is further increased by organizational and systemic factors, including staff experience variability, gaps in interprofessional communication, and the absence of standardized tools for real-time clinical supervision [7,8]. Not detecting patient deterioration at an early stage can result in potentially unnecessary adverse events, such as unplanned intensive care unit (ICU) admissions, longer hospitalizations, or even death [4,9].

Numerous studies show that any delay in detecting and responding to clinical deterioration increases morbidity, mortality, and healthcare costs, impacting both patient outcomes and the sustainability of healthcare systems [10,11].

To address these challenges, automated alert systems that leverage real-time data from electronic health records have been shown to enable earlier intervention [12]. These systems alert healthcare professionals when patients are at high risk. They do not replace the doctor’s judgment but support it, helping to monitor patients more effectively and enabling timely, targeted actions that can reduce mortality and improve outcomes [13].

Over the past 30 years, several early warning systems (EWS) have been proposed to convert vital signs and other physiological parameters into standardized scores capable of predicting patient deterioration. These systems aim to reduce reliance on subjective interpretation, ensuring that early warning signs are consistently recognized and appropriately acted upon [14]. Among these, the Early Warning Score [15] was one of the first to be developed, and its adoption led to the creation of variants, such as the Modified Early Warning Score, refined this approach by introducing thresholds and weighting schemes for key parameters [16].

These systems, combined with the measurement of patients’ vital signs, have become key tools in clinical practice to detect early deterioration and ensure immediate intervention [17].

A significant evolution occurred in 2012, when the Royal College of Physicians introduced the National Early Warning Score (NEWS) [18], derived from a modification of the ViEWS [19]. In 2017, this score was updated with the NEWS2 score for better identification of deterioration in specific groups of patients, particularly those with hypercapnic respiratory failure and sepsis, adding oxygen saturation thresholds and a specific alarm for patients with supplemental oxygen [20,21]. This score was designed as a system to assess the severity of an acute illness and is currently one of the most widely used early warning scoring systems [22,23]. This structured scoring system helps to identify early signs of patient deterioration, enabling timely intervention and intensification of care when necessary, while guiding decisions on patient monitoring [24].

NEWS has been validated in various clinical settings [18,25,26] and has demonstrated superior performance compared to other Early Warning Scores in predicting the combined outcome of cardiac arrest, unplanned ICU admission, or death within 24 h, offering significant potential to change patient outcomes [27]. Furthermore, NEWS has shown the highest agreement between predicted and observed outcomes, performing particularly well in different subgroups of patients, especially those with respiratory disease [28].

However, despite these recognized strengths and their widespread adoption, conventional Early Warning Score (EWS) systems such as NEWS2 exhibit significant limitations that can reduce their clinical effectiveness [24,29]. They can generate many false alarms, increasing the workload of clinicians without a real perceived benefit for nursing staff [30], while also failing to detect all deteriorating patients [31]. To overcome these limitations, the integration of machine learning and deep learning is emerging as a strategic solution to develop more accurate monitoring systems with reduced false alarm rates [32,33].

Nevertheless, while numerous studies have evaluated the effectiveness of the National Early Warning Score 2 (NEWS2) in predicting clinical deterioration in hospitalized patients, few have explored the potential of combining NEWS2 with other routine clinical data, such as the Barthel Index and the presence of a central venous catheter, to create a more comprehensive predictive model. Furthermore, most studies focus on risk prediction itself, without directly addressing how this information can be used to optimize nursing resource allocation. This model has a prognostic purpose, aiming to predict future events rather than diagnose a current condition.

This study aims to bridge this gap by developing and validating a predictive model, integrated with electronic health record (EHR) data, that combines NEWS2 with clinical and demographic variables readily available at admission. The model is intended for adult patients admitted to general medicine units. The objective is to identify patients who develop clinical deterioration and mortality within a general medicine unit, thereby enhancing the accuracy of risk stratification for this vulnerable population.

## 2. Material and Methods

This study adheres to the Transparent Reporting of a multivariable prediction model for Individual Prognosis Or Diagnosis (TRIPOD) 2015 guideline for retrospective studies [34]. The completed TRIPOD checklist is provided as supplementary material (Supplementary Materials).

### 2.1. Study Population

The study is a cohort study with retrospective data collection. Information was collected from all patients who entered the general medicine department of the University Hospital of Siena (Italy) from March 2022 to October 2023. The same patient may be present multiple times if he/she had multiple admissions, and in this case, all admissions were considered with updated information at the reference admission. Inclusion criteria were NEWS2 < 7 and age ≥ 18 years old. Patients with a NEWS2 ≥ 7 at admission, or those < 18 years old, were excluded from the analysis.

### 2.2. Electronic Health Record

Pleiade is the EHR system used by the Azienda Ospedaliero-Universitaria Senese. An EHR is a digital version of a patient’s chart, providing a real-time, patient-centered record that makes information available instantly and securely to authorized users. Pleiade is designed to ensure efficient data collection, digital signature authentication, and rapid access to vital parameters, pharmacological therapy, and clinical care pathways. Implemented across two hospital facilities, it serves over 200 UOC (hospital operating units), 2000 beds, and more than 10,000 registered users. As a full-web platform based on PHP, HTML5, and CSS3, Pleiade integrates seamlessly with all enterprise verticals, covering a broad range of territorial healthcare services. It supports secure archiving, digital preservation, and auto-generation of medical documentation. The system also manages patient privacy consent for clinical records and enables document transmission to the Tuscany Region’s Electronic Health Record 2.0. Pleiade incorporates multiple digital models and clinical-care pathways, offering predictive scoring and alert systems for early detection of critical biometric variations.

### 2.3. Ethical Aspects

Data were extracted from EHRs by authorized personnel other than the authors to whom data were shared in a fully anonymized manner; complete untraceability was ensured.

### 2.4. Variables

Age, sex, central venous catheter presence (CVC), revolving door (i.e., readmission within 60 days), Barthel Index, ReTos scale, and NEWS2 were collected at admission.

The ReTos scale is an instrument, validated for patients 65 years or older, used by the Tuscan Regional Health System (SSR) to identify the risk of falls in hospitalized patients. This scale was developed with the aim of creating a comprehensive tool that would resolve inconsistent and incomplete data collection resulting from the use of different scales such as Morse, Stratify, and Conley. The ReTos scale synthesizes the main risk factors of these instruments into three separate sections and adds a specific assessment of medication use. The final score is the sum of the sections, ranging from 0 (no risk) to 21 (high risk) [35]. The ReTos scale was also dichotomized using 7 as cut off [35].

The Barthel Index (BI) is a widely used ordinal scale designed to measure a patient’s degree of independence in performing essential Activities of Daily Living (ADLs). The Barthel Index is an ordinal scale used to measure the degree of independence of patients in performing essential ADLs. It assesses performance across ten fundamental items that encompass both self-care (e.g., feeding, dressing, and toilet use) and basic mobility. A cumulative score is calculated from these 10 items, ranging from 0 (indicating complete dependence) to 100 (representing complete independence) [36]. The Barthel Index was dichotomized using 60 as the cut off, identifying independent (Barthel index ≥ 60) and dependent (Barthel index < 60) patients [37].

The NEWS2 is scored from 0 to 20 and stratifies patients into three risk categories: low risk (≤4), medium risk (5–6), and high risk (≥7), with the latter group requiring continuous monitoring [22,38]. Only low-risk and medium-risk patients were present at admission due to the inclusion criteria.

The patients were then classified as HCR (High Clinical Risk) if NEWS2 became ≥ 7 during the hospitalization. Information regarding death during the hospital stay was also collected. The revolving door variable was dichotomized: ‘No’ for initial admission, ‘Yes’ for all subsequent admissions within 60 days.

### 2.5. Statistical Analysis

Descriptives were estimated. Categorical variables are reported as absolute frequencies and percentages, while quantitative variables are described using median and interquartile range [IQR]. We assessed the correlation between scales using Spearman’s rank correlation. Time-to-event analysis for HCR development and death was performed using Kaplan–Meier curves, comparing low-risk and medium-risk patient groups, with statistical significance assessed via the log-rank test.

Two separate logistic regression models were developed using a stepwise selection approach: one for predicting in-hospital mortality and one for identifying patients at risk of high clinical deterioration (HCR) during hospitalization.

Logistic regression was chosen for its interpretability and ability to provide well-calibrated probability estimates, which are essential for clinical implementation. While more complex machine learning techniques have been considered, logistic regression provides comparable performance with the advantage of greater transparency and ease of integration into existing EHR systems.

Only variables showing statistical significance in univariate analysis were included in the models. Missing data were handled as follows. The proportion of missing values for each predictor was assessed. Age, sex, NEWS2 at admission, and CVC status had no missing data. The Barthel Index had 10.9% missing values, which were imputed using median imputation. The ReTos scale was excluded from multivariable models due to a high proportion of missing values (>40%). The final model was run on a complete case dataset. The time window of the clinical deterioration and of the death was the entire duration of the hospital stay. Instead, predictors were measured at admission. We estimated the area under the ROC curve (AUC) with corresponding 95% confidence intervals. For each model, we selected two cut off points, one with a specificity of 90% and one with a sensitivity of 90%. The ReTos scale was included in the initial descriptive analysis to characterize the study population. However, due to a high proportion of missing values, it was excluded from the multivariable regression models to preserve the sample size and avoid potential biases associated with imputation of a large amount of data.

The dataset was split into a training sample (80%) and a validation sample (20%), ensuring the same proportion of HCR of the original dataset. The absolute value of the z-statistic for each model parameter was used to determine variable importance.

A *p*-value < 0.05 was considered statistically significant. All analyses were performed using R software version 4.4.2.

## 3. Results

### 3.1. Description of HCR Patients and Death Patients

**Figure 1:**
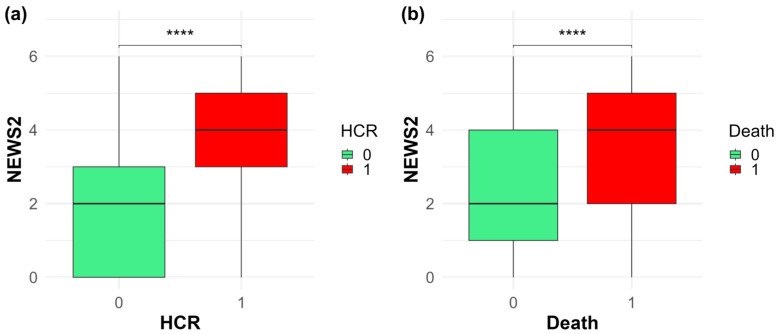
Boxplots comparing NEWS2 at admission between HCR (**a**) and death (**b**). Asterisks indicate statistical significance (**** *p* < 0.001).

The sample included a total of 2108 patients. Men and women were similarly represented in the sample (51.9% Male; 48.1% Female), and patients included in the study had a median age of 81 years (IQR: 70–87). The median length of hospital stay was 12.64 days (IQR: [8.52–19.46]). Approximately 29% of the patients (n = 612) developed HCR. The HCR group exhibited a significantly older age (*p* < 0.001) and greater CVC use (*p* < 0.001) compared to non-HCR patients. The Barthel Index was lower in patients with HCR (5 [0–45]), with 89.5% classified as dependent (Barthel < 60), compared to those who did not develop HCR (50 [10–92.5]), where 63.6% were dependent (*p* < 0.001). Patients who developed HCR also had significantly higher NEWS2 scores at admission (4 [3–5]), with 43% classified in medium risk, compared to those who did not develop HCR (2 [0–3], 11% medium risk) (Figure 1a) (see Table 1).

Taking into consideration the ReTos scale, Table 1, patients who worsened had a higher score at admission (7 [5–10]) than those who did not (6 [3–8]) (*p* < 0.001). Finally, no significant difference emerged in revolving door status (repeated hospitalizations) between HCR and non-HCR groups (*p* = 0.363) while mortality rates differed substantially between groups, with 32.8% of HCR patients dying compared to 3.9% in the non-HCR group (*p* < 0.001).

**Figure 2:**
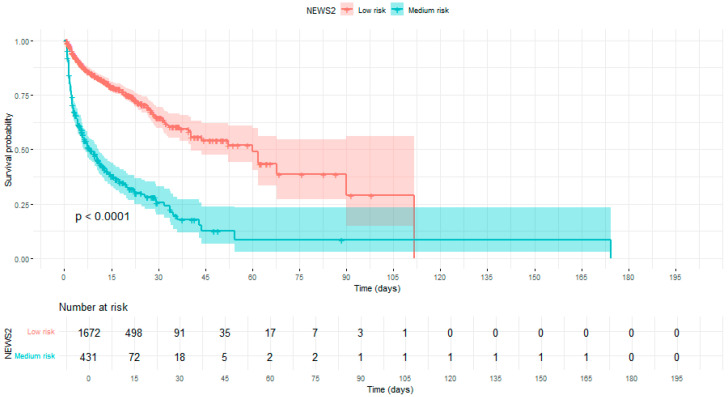
Comparison of time-to-events Kaplan–Meier between low-risk patients and medium-risk patients according to NEWS.

The median time to develop HCR was 4.33 days [IQR: 1.90–9.63], with a significant difference observed between patients with low risk (5.36 [2.41–10.94]) and medium risk (2.71 [1.44–7.36]) (*p* < 0.001). Figure 2 illustrates this difference with survival curves, further confirming the faster progression toward clinical deterioration in the medium-risk group compared to the low-risk group.

Deceased patients had a significantly higher median age than survivors (*p* < 0.001), with no significant differences between males and females (*p* = 0.33). No differences were found in the presence of CVCs (5.5% vs. 7.7%, *p* = 0.105). The proportion of patients with revolving door status was significantly higher among the deceased (*p* < 0.036). The deceased patients had a significantly lower Barthel Index at admission (5 [0–45]) compared to survivors (35 [5–80]) (*p* < 0.001). In addition, a higher proportion of deceased patients (87.4%) had a Barthel index < 60, compared to 68.9% of survivors. NEWS2 scores at admission were significantly higher in deceased patients (4 [2–5]) than in survivors (2 [1–4]) (*p* < 0.001) (Figure 1b). Moreover, 38.8% of deceased patients had medium risk in terms of NEWS2, compared to 17.9% of survivors. Conversely, no significant difference was observed in the ReTos scale (*p* = 0.168).

### 3.2. Comparison of NEWS2, Barthel, and ReTos with HCR and Death

The analysis revealed a weak positive correlation between NEWS2 and ReTos scores (r = 0.202, *p* < 0.001) and a moderate negative correlation between NEWS2 and the Barthel Index (r = −0.483, *p* < 0.001).

**Figure 3:**
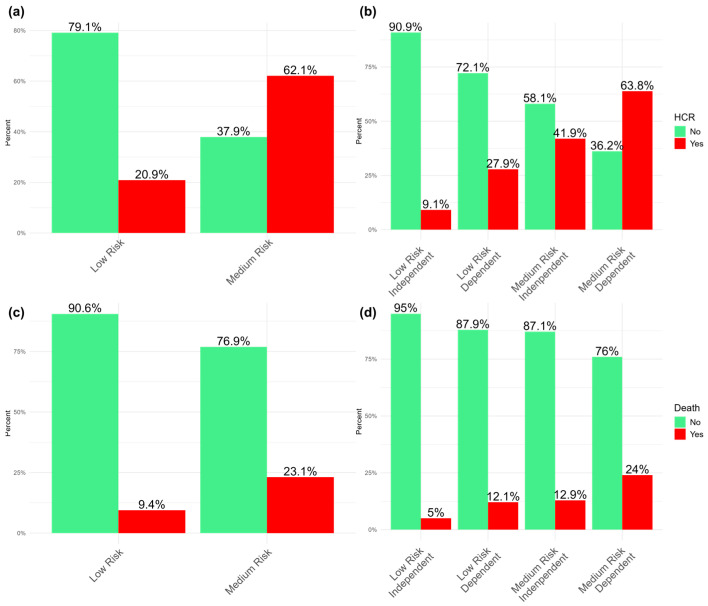
Probability of HCR or death, stratifying for NEWS2 and the Barthel Index. (**a**) Incidence of HCR according to NEWS2; (**b**) incidence of HCR stratified by NEWS2 and the Barthel Index; (**c**) incidence of death according to NEWS2; (**d**) incidence of death stratified by NEWS2 and the Barthel Index.

Of patients with a low risk (NEWS2 ≤ 4) at admission, 20.9% developed HCR during hospitalization. This percentage decreased to 9.1% if the patient was independent (Barthel ≥ 60), while it rose to 27% if not independent (Barthel < 60). Of patients who had a medium risk (NEWS2 = 5/6), 62.1% developed HCR during the hospital stay, and this percentage rose to 63.8% if the patient was also dependent, while it decreased to 41.9% if they were medium risk but independent (Figure 3).

Among patients with a low risk at admission, only 9.4% died; this percentage dropped further to 5% if the patient was independent, while it rose to 12.1% if the patient was dependent. Of patients with a medium risk, 23.1% died during the hospitalization; this percentage rose to 24% if the patient was also dependent, while it dropped to 12.9% if the patient was independent (Figure 3).

### 3.3. Predictive Models

Regarding the first model, with the HCR used as the dependent variable, the significant variable selected as the predictor in the stepwise procedure is shown in Table 2.

In particular, older age, presence of a CVC, a lower Barthel Index, and higher NEWS2 increased the likelihood of patients developing HCR during hospitalization.

**Figure 4:**
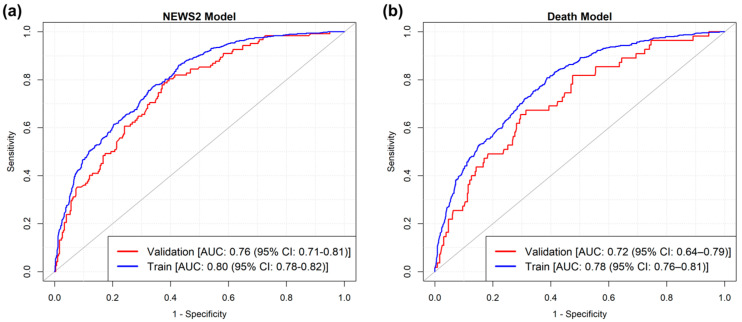
ROC of the logistic models according to training and validation samples: (**a**) NEWS2 model; (**b**) Death model.

The AUC of the model was 0.798 (95% CI: 0.776–0.820) in the training dataset and 0.758 (95% CI: 0.709–0.806) in the validation set, see Figure 4.

Two optimal cut-off values on the ROC curve were selected to classify patients’ risk of developing HCR during hospitalization. The first cut-off was 0.123, and the second was 0.522. Using these cutoff values, we stratified patients into three risk categories and assessed their predictive accuracy against actual HCR occurrence.

In the low-risk group, only 8.8% of patients developed HCR, while in the medium-risk group, this proportion increased to 31.3%. In the high-risk group, the proportion of patients who developed HCR was 61.4%.

The impact of the Barthel Index on the prediction of HCR risk was more pronounced in elderly patients (age > 80 years), suggesting that functional status is a particularly important factor to consider in this population.

For the second model, the significant variables selected as predictors for the dependent variable of mortality were age, the Barthel Index, and NEWS2. In particular, older age, lower Barthel Index, and higher NEWS2 score at admission increased the likelihood of patients dying during admission (Table 2).

To assess the performance of the model, the AUC was calculated on the validation set (0.716 (95% CI: 0.644–0.788)). To classify the risk of death for patients during hospitalization, two optimal cut-offs were selected on the ROC curve. The first cut-off was 0.059, and the second was 0.203. With this procedure, three risk categories were identified and then compared with actual deaths during hospitalization. In the low-risk group, only 4.7% of patients died, while in the medium-risk group, this proportion rose to 13.3%. In the high-risk group, the proportion of patients who died reached 29.4%.

Across both models, NEWS2 remained the most important variable (Table 2), followed by the Barthel Index, CVC (for the HCR model), and age.

## 4. Discussion

This study aimed to develop and validate a predictive model for early identification of hospital patients at high risk of clinical deterioration (HCR) and mortality during hospitalization using the NEWS2 score combined with additional clinical and demographic variables collected at hospital admission.

Interestingly, we observed a moderate negative correlation between NEWS2 and the Barthel Index (r = −0.483), indicating that higher levels of clinical deterioration are generally associated with greater functional dependency, i.e., lower Barthel. This finding highlights the importance of considering functional status in conjunction with physiological parameters when assessing risk.

This contrasts with Hodgson et al., where no significant correlation was reported between NEWS and functional status (r = −0.033) [39]. This discrepancy may arise from differences in study populations, as Hodgson et al. focused on residents living in care homes, a population with potentially different baseline functional abilities and trajectories of decline.

The results confirm that NEWS2 at admission is effective in measuring patient acuity and is associated with both the risk of clinical deterioration and mortality during the hospital stay. However, it is not sufficiently predictive on its own; in fact, models including age, CVC, and the Barthel Index significantly improved the model performance, highlighting the value of a multi-faceted approach. The predictive models developed in this article demonstrated a good predictive performance, achieving AUC values of 0.798 and 0.758 for HCR prediction in the training and validation sets, respectively. Likewise, the mortality model reached an AUC of 0.716 in the validation set. These results align with those of Haegdorens et al., who showed that, despite the effectiveness of NEWS in predicting adverse events, it becomes more predictive with the addition of the Nurse Intuition Patient Deterioration Scale [23], suggesting that incorporating nursing expertise can further refine risk stratification.

The definition of High Clinical Risk (HCR) as reaching a NEWS2 score ≥ 7 during hospitalization warrants further clarification. While this outcome inherently links to NEWS2 as a key predictor, which could lead to concerns about target leakage, our primary objective in this exploratory study was not to predict clinical deterioration in its broadest sense. Instead, the model aims to serve as an early warning tool, identifying patients at admission who are at high risk of reaching a critical NEWS2 threshold during their hospital stay.

This approach aligns with a realistic and operationally relevant use case, particularly in clinical settings where NEWS2 is already widely employed for triage and escalation. By providing a predictive signal before the critical NEWS2 threshold is met, the model offers clinicians a proactive time window for intervention. While we recognize that this approach inherits some of NEWS2′s intrinsic limitations, such as suboptimal sensitivity and specificity, it provides an actionable alert within current clinical workflows.

For future research, we recognize the importance of adopting more independent and robust clinical outcomes, such as unplanned Intensive Care Unit (ICU) transfers (UIT) or the need for critical care, as suggested by international consensus papers [40]. Such “hard” clinical outcomes are essential for a comprehensive and rigorous Health Technology Assessment (HTA), as they directly relate to patient safety, resource utilization, and healthcare costs. The current definition of HCR, while having operational utility in triage, will be reconsidered in future studies to align with broader and independent outcomes. The inclusion of Central Venous Catheter (CVC) presence at admission as a predictor also warrants further discussion. We acknowledge that CVC may serve as a proxy for higher baseline patient severity or previous care trajectories (e.g., recent ICU stays), potentially reflecting clinician-anticipated risk rather than an independent physiological deterioration. However, we chose to retain this variable as it represents readily available, objective data at the bedside, which clinicians often implicitly use to infer a patient’s underlying complexity or fragility. Our inclusion criteria (NEWS2 < 7 at admission) helped to exclude patients already in extremely critical conditions who would typically be directly admitted to the ICU, thereby mitigating some of the bias from direct ICU admissions with pre-existing CVCs.

While retaining CVC data reflects real-world clinical information flow, we acknowledge that it could potentially limit the generalizability of the model. Therefore, to address this more rigorously, future research will include specific subgroup analyses (e.g., comparing model performance in cohorts with and without CVC, or in patients transferred from ICU versus directly admitted to the ward) and ablation studies (evaluating model performance with and without the CVC variable). Such comprehensive analyses are paramount for building robust evidence. From an HTA perspective, further investigation into the independent contribution of variables like CVC will be crucial to fully ascertain the model’s value proposition and ensure that any predictive benefits are truly derived from novel clinical signals rather than merely reflecting pre-existing severity or care pathways.

By comparing the results obtained from the model with those in Figure 3c, a significant improvement in risk classification was observed. The proportion of patients classified as low risk at admission who subsequently died during hospitalization decreased by 4.4%. Similarly, mortality among medium-risk patients was reduced by 9.8%. This suggests that the proposed model is associated with more accurate risk stratification, potentially enabling more targeted clinical interventions. A more accurate prediction of deterioration allows for intensified monitoring of high-risk patients, which could support more timely responses and help mitigate negative outcomes. Our results suggest that incorporating the Barthel Index into the assessment of clinical deterioration risk may improve the accuracy of prediction, especially in elderly patients. Future studies should evaluate the effectiveness of this integration in a real-world clinical setting, comparing the results with those obtained using NEWS2 alone. Furthermore, our research opens the door to exploring other variables, such as frailty and cognitive status, which may further enhance risk prediction. The inclusion of frailty, for example, could help to identify patients who are more vulnerable to adverse outcomes, even with relatively stable physiological parameters.

Although NEWS2 is an easy-to-use tool for nurses and useful for identifying deterioration, it has some limitations [24,29]. Based on standardized physiological parameters, it can generate false alarms or fail to detect real deterioration, especially in elderly individuals or those with respiratory or cognitive issues, potentially leading to excessive unnecessary interventions or delays in care, thereby increasing the nurses’ workload without any real benefit [30,41].

Compared to more complex scoring systems such as APACHE II [42], NEWS2 is easier to use, allowing for rapid patient assessment. However, this can limit its predictive accuracy compared to scores that are more robust but at the same time more difficult to use in a real-world setting [43,44,45]. Our model, on the other hand, aims to find a balance between the practicality of NEWS2 and the analytical capabilities of more complex systems such as APACHE II, seeking to improve the predictive capacity of NEWS2 alone.

Therefore, the use of a more comprehensive model, incorporating functional and socio-clinical data, could help to mitigate these limitations, while also improving nurse–patient allocation by aligning care with individual patient needs.

Despite these limitations, NEWS2 remains a widely adopted tool. Notably, its use is mandated by regional authorities in Tuscany [46], which ensured the availability of complete NEWS2 data for all patients included in this study.

This system has the potential to support decision making, enabling more precise identification of patients who need intensified monitoring or timely interventions, while contributing to a more efficient distribution of the nursing workload.

A potential next step for this model would be its integration with the hospital’s EHR system, enabling nurses to perform real-time risk assessments at the bedside and facilitating more informed decision-making regarding patient monitoring and resource allocation.

Furthermore, categorizing patients into low-, medium-, and high-risk groups using optimized thresholds provides a practical method for clinical risk stratification. The integration of predictive models into the clinical flow enables more efficient allocation of resources to the most critical patients. In addition, this system can have a positive impact on nursing staff, helping to reduce stress levels related to work overload and the burden of daily decision-making, allowing them to focus more on the most critical patients and making work more manageable [47,48].

A crucial aspect of a prediction model’s performance is its calibration, i.e., the agreement between predicted and actual observed risk probabilities. While logistic regression was chosen for its interpretability and its ability to generate probability estimates, a formal calibration analysis (e.g., via calibration plots or Hosmer–Lemeshow test) was not performed in this initial exploratory study. Our primary objective was to assess whether the integration of additional clinical variables could improve the discriminatory power of NEWS2 alone within a single-center cohort.

The absence of presented calibration plots is a recognized limitation of this study. However, we commit to making calibration analysis an integral and prominent part of subsequent external validation phases of the model. Only a well-calibrated model can provide accurate probability estimates necessary for informed clinical decision-making. Such robust calibration is also crucial for HTA studies, helping ensure appropriate resource allocation and patient safety. Beyond calibration, this study presents several inherent limitations stemming from its retrospective design. Recognizing that retrospective studies, like the present one, are classified as early stage development for predictive models, we acknowledge their inherent limitations. Such studies use retrospective data, which may be subject to biases and limits the generalizability of findings.

A critical concern for any predictive model, especially those derived from complex retrospective data, is overfitting, which can severely compromise its generalizability and lead to poor performance in real-world prospective settings and critical degradation in performance upon implementation [49,50,51]. While a formal sensitivity analysis comparing different imputation strategies was not conducted for this exploratory phase, we acknowledge its importance for assessing the robustness of results to missing data handling, as high rates of missing input data can also contribute to performance degradation. The execution of more in-depth sensitivity analyses, for example, comparing the impact of different imputation strategies, will be an essential component of future external validation phases of the model. Therefore, testing the model on completely new, prospective data is essential to confirm its real-world utility. Another limitation stems from the exclusion of certain variables (e.g., ReTos scale) due to the high number of missing values; furthermore, as this scale was developed and evaluated internally by the Tuscany region, its generalizability is less established compared to the variables selected for our model. However, we intentionally designed a simple model that relies on a few variables for which data were largely complete. While the Barthel Index required imputation, this was performed on a very small number of observations, minimizing its potential impact. Our model focuses on predicting HCR and mortality but does not directly address other important outcomes, such as length of stay or readmission rates.

## 5. Conclusions

In conclusion, this study highlights the potential value of integrating the Barthel Index and other clinical and demographic variables with NEWS2 to predict patient risk profiles during hospitalization. The developed models demonstrate that integrating a measure of functional dependency is associated with significantly improved risk stratification compared to using physiological parameters alone. The proposed model offers a tool to support clinical decision-making, potentially allowing for a more accurate and timely identification of patients who may require intensified monitoring or intervention. However, given the retrospective nature of this study, direct causal claims regarding improvements in patient outcomes cannot be made. Future research must focus on the prospective validation of this model in diverse clinical settings and on rigorously evaluating its impact on care processes and patient outcomes through interventional studies. Such evidence is critical for a comprehensive Health Technology Assessment of this predictive tool.

## Figures and Tables

**Table 1:** Characteristics of the 2108 patients included in the study and comparison between HCR and death.

Variabile	NEWS ≤ 7	NEWS ≥ 7	*p*	Survived	Died	*p*
n	1496	612		1848	260	
Male	769 (51.4)	324 (52.9)	0.553	966 (52.3)	127 (48.8)	0.333
Cvc	59 (3.9)	62 (10.1)	<0.001	101 (5.5)	20 (7.7)	0.193
Revolving Door	113 (7.6)	62 (10.1)	0.063	144 (7.8)	31 (11.9)	0.032
Age	79 [67, 86]	84 [75, 89]	<0.001	80 [69, 87]	85.00 [76, 88]	<0.001
Barthel	50 [10, 92.5]	5 [0, 45]	<0.001	35.00 [5, 80]	5.00 [0, 45]	<0.001
NEWS2	2 [0, 3]	4 [3, 5]	<0.001	2 [1, 4]	4 [2, 5]	<0.001
ReTos	6 [3, 8]	7.00 [5, 10]	<0.001	6 [4, 9]	7 [4, 9]	0.168
Died	59 (3.9)	201 (32.8)	<0.001			
High Risk				411 (22.2)	201 (77.3)	<0.001

**Table 2:** Univariate and multivariate odds ratios of HCR and mortality.

	HCR			
	OR Univariate	OR Multivariate	*p* Value	Variable Importance
CVC	3.04 (2.00–4.67)	3.81 (2.34–6.27)	<0.001	5.33
Age	1.038 (1.03–1.05)	1.02 (1.01–1.03)	<0.001	4.00
Barthel	0.976 (0.97–0.98)	0.988 (0.98–0.99)	<0.001	5.49
NEWS2	1.72 (1.61–1.84)	1.57 (1.46–1.69)	<0.001	12.50
		Death		
	OR Univariate	OR Multivariate	*p* Value	Variable Importance
Age	1.03 (1.02–1.05)	1.018 (1.00–1.03)	0.008	2.64
Barthel	0.984 (0.98–0.99)	0.99 (0.99–1)	0.002	3.11
NEWS2	1.336 (1.24–1.44)	1.228 (1.13–1.34)	<0.001	4.72

## Data Availability

The datasets analyzed during the current study are available from the corresponding author upon reasonable request.

## Supplementary Materials

The following supporting information can be downloaded at: https://www.mdpi.com/article/10.3390/medicina61091543/s1, Table S1: TRIPOD Checklist.

## Abbreviations

The following abbreviations are used in this manuscript:

NEWS2	The National Early Warning Score 2
EWS	Early warning systems
ICU	Intensive care unit
CVC	Central venous catheter
HCR	High clinical risk
AUC	Area under the curve
EHR	Electronic health record
UOC	Hospital operating units
ADLs	Electronic health record
IQR	Interquartile range
